# Urdu-Language Translation and Validation of the Ask Suicide-Screening Questions (ASQ) Tool: A Focus on Connotation and Context

**DOI:** 10.7759/cureus.65763

**Published:** 2024-07-30

**Authors:** Khalid I Afzal, Aleena Cheema, Hassan Cheema, Annabelle Mournet, August Wei, Areeha Khalid, Ritika Merai, Maryland Pao, Lisa Horowitz

**Affiliations:** 1 Psychiatry and Behavioral Neuroscience, The University of Chicago Medicine, Chicago, USA; 2 Internal Medicine, Services Institute of Medical Sciences (SIMS), Lahore, PAK; 3 Office of the Clinical Director, Intramural Research Program, National Institute of Mental Health, Bethesda, USA; 4 Graduate Medical Education, Midwestern University Chicago College of Osteopathic Medicine, Downers Grove, USA

**Keywords:** ask suicide-screening questions (asq), questionnaire, screening, suicide risk, urdu

## Abstract

Background

Suicide is a significant cause of death in the world, and Pakistan, a low- and middle-income country, is no exception. Despite the increasing number of suicides, Pakistan does not have a validated suicide risk screening tool to identify suicide risk in the national language, Urdu, accurately. This study aims to translate and validate the Ask Suicide-Screening Questions (ASQ) tool into Urdu for suicide risk screening in Pakistan.

Methodology

We conducted this study at the Services Institute of Medical Sciences (SIMS), a large teaching hospital in Lahore, Pakistan, after receiving the approval of the SIMS Institutional Review Board. The study used a cross-sectional instrument validation study design. The inclusion criteria were youth and adults of both sexes aged 15-45 years, with an ability to understand, speak, read, and write in the Urdu language, who had no cognitive or intellectual limitation to consenting, and who were medically stable to participate. Exclusion criteria included any medical, physical, or cognitive unstable condition to consent or participate. We enrolled 300 participants in our convenience sample from the emergency department (ED), inpatient, and outpatient settings. The ASQ and the ASQ Brief Suicide Safety Assessment (BSSA) were translated and back-translated by Urdu language experts and modified to accommodate cultural and linguistic nuances. The clinician-administered BSSA Urdu version was used as a standard criterion to validate the ASQ by comparing the ASQ-Urdu responses vs. BSSA-Urdu responses. RStudio (version 2023.09.1+494) was used for statistical analyses

Results

The sample had an enrollment rate of 99.7% (300/301). The sample was 52% female (158/300); the mean age was 27.1 years (SD = 9.4), the overall screen-positive rate was 41.7% (125/300), and 9.3% (28/300) of the participants endorsed a past suicide attempt. In our sample, 35.9% (33/92) of outpatients, 32.2% (19/59) of inpatients, and 49.0% (73/149) of ED patients screened positive on the Urdu ASQ. The screen-positive rate was 16.9% (10/59) for participants aged 17 years and younger, 40.7% (35/86) for participants aged 18 to 25 years, and 51.6% (80/155) for participants aged 26 years and older. Compared to the criterion standard clinician-administered assessment, the Urdu ASQ had a sensitivity of 94.2% (95% confidence interval (CI) = 85.8%-98.4%), a specificity of 73.9% (95% CI = 67.7%-79.5%), a negative predictive value of 97.7% (95% CI = 94.2%-99.1%), and a positive predictive value of 52.0% (95% CI = 46.4%-57.6%).

Conclusions

The Urdu ASQ has strong psychometric properties, allowing healthcare professionals in Pakistan and worldwide with Urdu-speaking diaspora to identify individuals at risk for suicide efficiently. Utilizing cultural contexts in adapted screening tools improves the accuracy of suicide detection by ensuring that the tools are relevant, sensitive, and respectful to the cultural context of the individuals being assessed. High screen-positive rates in our pilot study underscore the need for early detection and intervention of suicide as a major global public health problem.

## Introduction

Suicide is a global public health crisis. The World Health Organization estimates that over 700,000 people die by suicide yearly [1]. Overall, 77% of global suicides occur in low- and middle-income countries (LMICs) [1]. Pakistan, an LMIC, is the fifth most populous country in the world, with an estimated suicide rate of 9.8 per 100,000 in 2019 [2], translating to 15 to 35 people ending their lives daily. Most suicides occur in individuals 30 years old or younger, with a 3:1 male-to-female ratio of completed suicide. About 87% of those who committed suicide were identified as married compared to 10% being single. The preferred method is poisonous substance ingestion, followed by hanging and firearms [3]. However, the lack of vital registrations or a central mortality database limits the accuracy of suicide figures in Pakistan. Religious and cultural condemnation of suicide and self-harm may lead to further stigma and under-reporting of suicidal behaviors [4].

Historically, suicide or suicide attempts were considered criminal offenses under the Pakistan Penal Code 309 of the Criminal Procurement Act, punishable by imprisonment and/or subject to a financial penalty of up to Rs 10,000 (roughly $36) before the Pakistan Senate passed a bill to decriminalize suicide in 2018 [5]. Despite these changes, the public has yet to accept treatment for suicide risk and implement proactive suicide prevention measures [6]. Suicide risk is not routinely identified or assessed in medical settings except to address a suicide-related presenting complaint or as part of a psychiatric evaluation [4]. Consequently, many individuals with suicide risk may pass through healthcare systems in Pakistan undetected.

Urdu is the official language among the 72 plus languages spoken in Pakistan [7]. Overall, 80% of Pakistan’s population (estimated >250 million) understands Urdu or speaks it as a second language. About 7%, or about 17 million, are considered “Urdu speaking” or have Urdu as their first language [7]. Urdu is spoken by more than 100 million people across the Indian subcontinent and expatriates living in the Middle East, Europe, and North America [7]. English is the co-official language of Pakistan, as it is in other formal British or American colonies such as India, Kenya, or the Philippines, but it is not as widely spoken by its residents as Urdu. To our knowledge, there is no validated suicide risk screening instrument available in Urdu, the national language of Pakistan, to aid clinicians in accurately detecting suicide risk in patients.

Urdu belongs to the Indo-Aryan group within the Indo-European family of languages, and its grammar draws heavily from Arabic, Persian, and Turkic languages [8]. As opposed to English, written from left to right, Urdu uses the Perso-Arabic script, written from right to left [7]. The associated connotation and context heavily influence spoken Urdu. The Merriam-Webster dictionary defines “connotation” as the “implicit or hidden meaning.” For example, “home” connotes “safety,” “love,” “belonging,” etc. [9,10]. Contexts could be divided into physical (location, timing, setting, etc.), epistemic (shared background knowledge), linguistic (already been said), social (relationship between the speaker and the listener), historical, and cultural [10]. Uzair et al. further suggest that Urdu is a highly inflected language where words are inflected to express gender, number, person, and case [9].

Problems in translating a survey or questionnaire have long been recognized [11]. Ervin and Bower argue that a direct or verbatim translation of questions may fail to convey the meaning of the original items [11]. They identified several reasons that could potentially distort the translation, such as differences in the meaning of words, syntactical contexts, and the cultural contexts of the readers or the listeners. Factors such as the lexical meanings of objects in different cultures may have a larger range of referents than in another, homonyms or single words having several meanings (e.g., pen), affective and figurative meanings of words (hope or expectation for prisoners), and some words may not exist in another language and are untranslatable were also highlighted. Grammatical meaning, stylistic factors, problems due to dialects, and the difference between spoken and written language could further complicate the matter [11].

The National Institute of Mental Health (NIMH) developed the evidence-based suicide risk screening Ask Suicide-Screening Questions (ASQ) tool in 2008 to assess suicide risk initially in young patients in medical settings to detect individuals at risk for suicide rapidly [12]. A panel of mental health clinicians, health services researchers, and survey methodologists adapted 17 candidate suicide screening questions based on youth suicide risk factors such as previous suicide attempt history, suicidal ideation, depression, hopelessness, substance abuse, and social isolation. By calculating sensitivity, specificity, positive predictive value (PPV), and negative predictive value (NPV), five questions were included in the ASQ scale by selecting the best-fitting models based on clinical and statistical significance [12]. Since then, it has been validated for all ages [13]. The ASQ is validated in the pediatric emergency department (ED), inpatient/surgical settings [14], and outpatient primary care/specialty clinics [15], as well as in adult inpatient settings [13,16]. Moreover, the ASQ is publicly available, translated into over 23 languages, and validated in Spanish, Japanese, and Nepali, to name a few [17]. Other translations have not been validated yet for the native language-speaking population. This study aims to translate and validate the culturally responsive Urdu version of the ASQ to ensure fairness, accuracy, and cultural sensitivity in clinical and educational applications across groups, leading to better outcomes and support. It also aims to provide preliminary evidence on the acceptability and accuracy of the Urdu ASQ to detect suicide risk in Urdu-speaking/receptive medical settings in an urban medical center in Pakistan.

## Materials and methods

Study design

We partnered with the Services Institute of Medical Sciences (SIMS), a 1,196-bed teaching hospital in Lahore, Pakistan. The SIMS Institutional Review Board approved the study, and the SIMS hospital administration provided administrative support. Three SIMS medical school graduates were recruited and trained during their intern year to collect data for the study. The training included the study design, data confidentiality, patient approach, and consenting (written consent from patients aged 18 years or older and written assent from patients aged 15-17 years). The researchers received two-hour video-based live instruction via Zoom to learn the Urdu ASQ and Brief Suicide Safety Assessment (BSSA) Urdu administration and preparation of post-study mental health referral resources [18]. A medical attending was present at each location to provide support for patients with positive suicide risk screens. The SIMS psychiatry department agreed to provide a complete psychiatric evaluation for patients with immediate safety concerns or suicidality. The COVID-19 pandemic lockdown ensued during the project launch, delaying data collection by a few months. The study was completed in person between May and July 2020 in the ED, inpatient, and outpatient settings. All patients received outpatient resources, including contact information from the SIMS psychiatry department for counseling services. Participation in the study was voluntary, and the patients received no compensation. The inclusion criteria were (1) youth and adults aged 15-45 years; (2) all sexes; (3) ability to understand, speak, read, and write in the Urdu language; (4) cognitive and intellectual capacity to consent for the study; and (5) medically stable to participate. The exclusion criteria were any medical, physical, or cognitive unstable condition to consent or participate.

ASQ translation

The ASQ can be administered in under one minute and comprises the following four binary (Yes/No) questions: (1) In the past few weeks, have you wished you were dead? (2) In the past few weeks, have you felt that you or your family would be better off if you were dead? (3) In the past week, have you been having thoughts about killing yourself? (4) Have you ever tried to kill yourself? If the patient answers Yes to any of the above, the patient is asked the following acuity question: (5) Are you having thoughts of killing yourself right now?

We completed the ASQ Urdu translation in three steps. In the first step, three native Urdu-speaking researchers completed a verbatim translation of the ASQ into Urdu. The accuracy of the translation was confirmed by back-translation into English by another native Urdu speaker utilizing the criteria for semantic equivalence, which compared meaning or predictions of similar responses to original or translated versions. The discrepancy between translators was resolved by consulting an Urdu language expert with a master’s in Urdu language and teaching experience who was not directly involved with the research team.

Second, we administered the ASQ to 25 randomly selected adult patients (12 from outpatient, and 13 from ED) and gathered their feedback to determine the cultural acceptability of the Urdu translation. We wished to gain insight from a small, culturally, and demographically inclusive sample that could inform modifications and improvements to the screening tool and study design, leading to subsequent success when used in larger samples. Most participants raised two concerns about the first ASQ question:

Original English: In the past few weeks, have you wished you were dead?

Urdu translation: پچھلے چند ہفتوں میں کیا آ پ کو مرنے کی خواہش ہوئی ہے؟

First, the use of “wish” (“Khwahish” in Urdu) in Urdu connotes a positive experience (i.e., a birthday wish, wishing someone good luck or happiness). Second, asking about being “dead” (“marnay ki” or “marna” in Urdu) was off-putting to many participants due to the stigma surrounding talking about suicide in Pakistan. Word-by-word Urdu translation was grammatically accurate but was culturally questionable.

After discussions with the research team, we modified the first ASQ question to accommodate the cultural connotation and context of the Urdu language. We replaced the word “Khwahish” (“wish”) with four Urdu words, “khyal aya kay kaash (thought of that…).” “Kaash” is the closest to “wish” in English and lacks the positive connotation of “Khwahish.” “Kaash” is neutral in connotation and could be positive or negative according to the context. Additionally, we substituted “marnay ki or marna” (“dead”) with “zinda na hotay” (“not being alive”) to circumvent the cultural stigma of asking about “death. By replacing “wish you were dead” with “thoughts of not being alive,” we avoided the objection that inquiring about “death” was a “bad omen” and could superstitiously summon a tragedy [19].

The modified question was: In the past few weeks, did you have thoughts of not being alive?

The Urdu translation became: (see Appendices) پچھلے چند ہفتوں میں آ پ کوخیال آیا کہ کاش آپ زندہ نہ ہوتے؟

In the third step, we back-translated with native Urdu speakers to confirm the accuracy of the modified question before administering the new version to another group of volunteers in the ED and outpatient setting. About 20 volunteers out of 25 reported no further concerns. After receiving positive feedback on the cultural acceptance of all ASQ questions, we finalized the translation. The ASQ BSSA was also translated and back-translated into Urdu for this study.

Screening and assessment procedures

A patient screens positive on the ASQ if they answer “Yes” to at least one of the first four questions. Patients who screen positive are asked the fifth question to determine acute versus non-acute risk. We used clinician assessment as the criterion standard; clinicians utilized the Urdu version of the ASQ BSSA to guide their assessment [18,20] (Appendices). The BSSA, available in adult and pediatric, site-specific versions [18], is a series of interview-style questions designed as an intermediate-risk stratification guide [21]. The BSSA is typically administered in 10 to 15 minutes by a trained clinician and explores suicide risk factors (e.g., suicidal ideation, specific suicide plan, previous suicide attempts, or self-injury; a history of depression, anxiety, impulsivity/recklessness, hopelessness, irritability, substance and alcohol use, any recent stressors) and protective factors (e.g., support networks, how the patient could stay safe, reason for living). All ASQ and site-specific BSSA materials can be found on the ASQ Toolkit website at https://www.nimh.nih.gov/ASQ [18].

After obtaining consent (written consent from adult patients, written assent from youth 15-17 year olds, and written consent from youth’s parents/guardians), physician researchers administered the Urdu ASQ to each patient. Immediately after, another trained physician-researcher who was blind to the initial ASQ result approached the same patient and completed a brief suicide safety assessment using the Urdu version of the ASQ BSSA as a guide. Agreement rates between the Urdu ASQ and the clinician assessment were calculated.

Statistical analysis

RStudio (Version 2023.09.1+494) was used for statistical analyses. A p-value of less than 0.05 was considered the criterion for statistical significance. Adjustments for multiple comparisons were not made, given the utilization of a few analyses. Descriptive statistics and univariate and multivariate analyses are reported regarding screen-positive rates on the ASQ by demographic variables and medical setting. Chi-square tests were performed to examine differences in rates by group. A clinician-administered BSSA was considered the standard criterion. We calculated the proportion of true cases of suicide risk (as determined by the clinician assessment) who screened positive on the Urdu ASQ (sensitivity) and the percentage of true non-cases out of those who were identified to be negative by clinician assessment (specificity). We also calculated the proportion of true negatives out of those who screened negative on the Urdu ASQ (NPV) and the proportion of true positives out of those who screened positive on the Urdu ASQ (PPV).

## Results

The researchers approached 301 patients, aged 15 to 45 years, in the ED (149 patients), inpatient surgical or medical units (59 patients), and outpatient clinics (92 patients) (Table 1). All who were approached except for one individual consented to participate in the study. The sample was 52.7% female (158/300) and had a mean age of 27.1 years (range = 15-45; SD = 9.4). The screen-positive rate was 41.7% (125/300), and a total of 28 participants endorsed a past suicide attempt (9.3%; 28/300). The screen-positive rates by settings were as follows: 35.9% (33/92) for outpatients, 32.2% (19/59) for inpatients, and 49.0% (73/149) for ED patients (χ² = 6.7; p = 0.034). For females, the screen-positive rate was 45.6% (72/158), and for males, the screen-positive rate was 37.3% (53/142; χ² = 1.7; p = 0.184). For participants 17 years and younger, the screen-positive rate was 16.9% (10/59), 18 to 25 years was 40.7% (35/86), and 26 years and older was 51.6% (80/155; χ² = 21.2; p < 0.001) (Table 1). Overall, 23% of participants were identified as being at risk for suicide on clinician assessment (69/300). Compared to the criterion standard clinician-administered assessment, the Urdu ASQ had a sensitivity of 94.2% (95% confidence interval (CI) = 85.8%-98.4%), a specificity of 73.9% (95% CI = 67.7%-79.5%), an NPV of 97.7% (95% CI = 94.2%-99.1%), and a PPV of 52.0% (95% CI = 46.4%-57.6%).

**Table 1:** ASQ screen-positive rates by demographic groups. ASQ = Ask Suicide-Screening Questions; ED = emergency department

	Demographic group, N (%), mean (SD), range	ASQ screen-positive rate, N (%)	Significance
Gender			χ² = 1.7, p = 0.184
Female	158 (52.7%)	72 (45.6%)	
Male	142 (47.3%)	53 (37.3%)	
Age	21.7 (9.4), 15–45		χ² = 21.2, p = 0.001
15–17	59 (19.7%)	10 (16.9%)	
18–25	86 (28.7%	35 (40.7%)	
26+	155 (51.7%)	80 (51.6%)	
Setting			χ² = 6.7, p = 0.034
Outpatient	92 (30.7%)	33 (35.9%)	
Inpatient	59 (19.7%)	19 (32.2%)	
ED	149 (49.7%)	73 (49%)	

## Discussion

The translated scale, Urdu ASQ, demonstrated strong psychometric properties for identifying patients at risk for suicide in urban medical settings in Pakistan. Compared to the criterion standard clinician-administered assessment, it had a sensitivity of 94.2% and specificity of 73.9%, consistent with high psychometric properties found in previous clinically administered scale ASQ validation studies conducted among English-speaking participants [12-15].

Clinically administered scales are administered by trained clinicians or healthcare professionals who interpret the questions, ensure the respondent understands them, and may observe and interpret responses beyond what is explicitly stated [21]. On the other hand, the translated scale is adapted from one language to another, with the primary focus on maintaining the same meaning and psychometric properties as the original scale. Both clinically administered and translated scales measure specific constructs and undergo rigorous processes (pilot testing, statistical analysis, and expert view) to improve reliability and validity. The translated scales highlight cross-cultural validation studies and comparisons with the original scale’s results.

Clinically administered scales emphasize clinical applicability and professional interpretation, while translated scales focus on maintaining the accuracy and cultural relevance of the original tool across different languages [21].

Consistent with previous implementations of suicide risk screening, the screen-positive rates were approximately similar in the medical outpatient and inpatient settings and comparatively higher in the ED, likely related to the acuity of clinical presentation [22]. However, the high screen-positive rate and lifetime suicide attempt prevalence were unexpectedly high across medical settings. Recent studies have indicated many situational stressors (e.g., domestic conflicts, financial struggles, love/marriage failures, exam failure, sexual assaults, forced marriages, court trials, and bereavement) as common characteristics of suicide attempters in Pakistan [23]. The timing of the study at the peak of the COVID-19 pandemic-related lockdown possibly contributed to the high suicide rate. Our sample represented many individuals of low socioeconomic status, such as daily wage workers or individuals whose income depended on selling items such as fruits or vegetables in stalls [24]. The COVID-19 lockdown severely impacted them as the public was mandated to stay indoors or work remotely. Furthermore, the closure or Zoom transitioning of educational institutions further reduced the traffic and affected their income [25].

In our sample, all but one approached participant consented to participate in the study, likely related to the acuity of their medical condition. This participation rate was unusually high compared to previous ASQ validation studies [13,15,26,27]. We assume that Pakistan’s prevailing cultural hierarchical system that prioritizes the authority of doctors influenced the high participation rate in our study [28]. Pakistani patients often opt to delegate decision-making to the doctor or other family members, trusting them to make the best decision for them [29].

The high prevalence of suicidal ideation and attempts among the sample in the study highlights the need for validated universal suicide risk screening and assessment tools in non-English native languages. The study also reflects the need for establishing a clinical pathway in LMIC medical settings to stratify positive suicide risk screens by the use of validated risk stratification tools such as youth suicide clinical pathways [20,30] and the ASQ BSSA, limiting the overuse of already burdened healthcare systems. Teaching providers immediate interventions, such as making safety plans with patients and conducting lethal means safety counseling, will help make the identification of suicide risks in LMIC medical settings more feasible.

Utilizing cultural context in adapted screening tools improves the accuracy of suicide detection by ensuring that the tools are relevant, sensitive, and respectful to the cultural context of the individuals being assessed. Furthermore, this facilitates better communication, enhancing risk factor identification, and more effective prevention and intervention efforts. Cultural adaptations may improve individual acceptance, leading to an increased response rate and improved reliability of measures.

Limitations

Our study has some limitations. First, it is unclear whether the non-verbatim modifications to the Urdu ASQ translation influenced the high screen-positive rate (i.e., under or over-detecting suicide risk). Second, the study was conducted in an urban center and enrolled a convenience sample with high Urdu literacy rates, limiting generalizability to a wider population of rural residents with varying education levels. Third, the authors are sensitive to the need for more culturally sensitive ASQ translations that are tailored to the regional languages across Pakistan’s five provinces: Punjab, Sindh, Balochistan, Khyber Pakhtunkhwa, and Gilgit-Baltistan [5].

## Conclusions

This study found that the Urdu ASQ has strong psychometric properties, allowing healthcare professionals in Pakistan and worldwide for the Urdu-speaking diaspora to accurately and rapidly identify individuals at risk for suicide. Researchers must consider cultural and linguistic nuances while translating instruments to Urdu, as a verbatim translation may not be culturally acceptable or responsive. With the rapidly rising suicide rate in Pakistan, a validated, culturally informed suicide scale will provide clinicians with a tool to assess safety in busy emergency rooms or outpatient practices efficiently. Future studies should replicate findings in other cities, districts, and rural health centers in Pakistan to determine the universal acceptability of the Urdu ASQ. The data collection must ensure a comprehensive representation of the target population to draw meaningful conclusions, and the results could guide future refinement of the translation based on new insights and feedback.

## Figures and Tables

**Figure 1:**
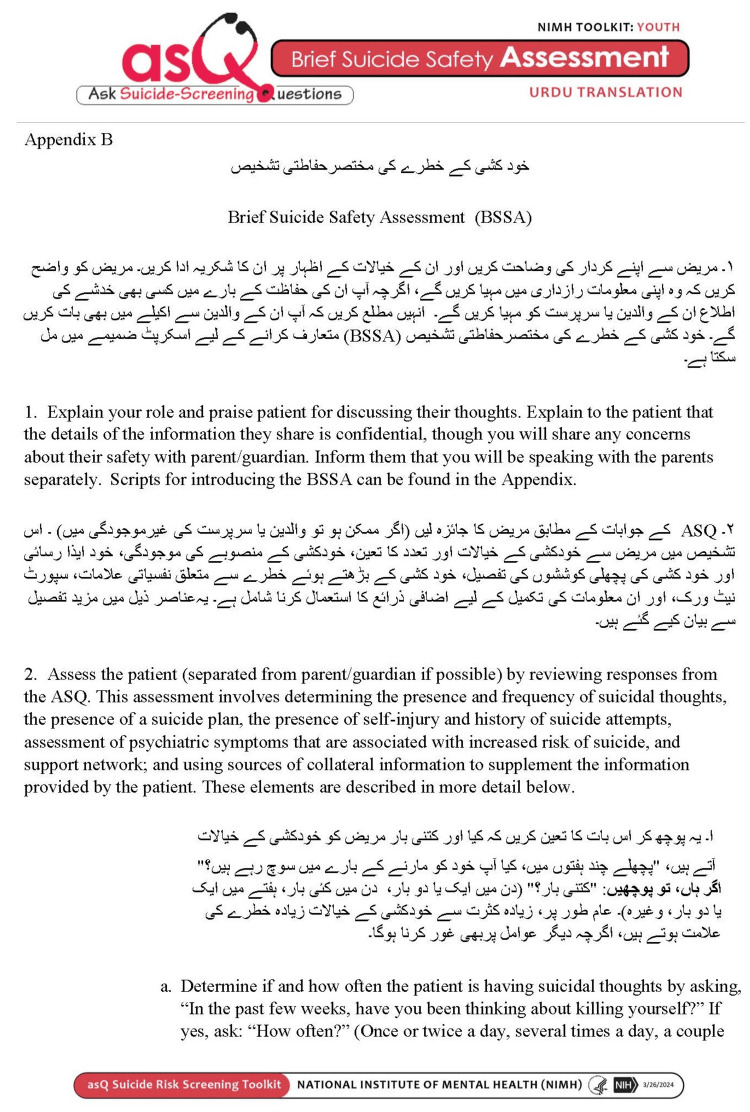
Brief Suicide Safety Assessment (BSSA) Urdu certified translation page 1.

**Figure 2:**
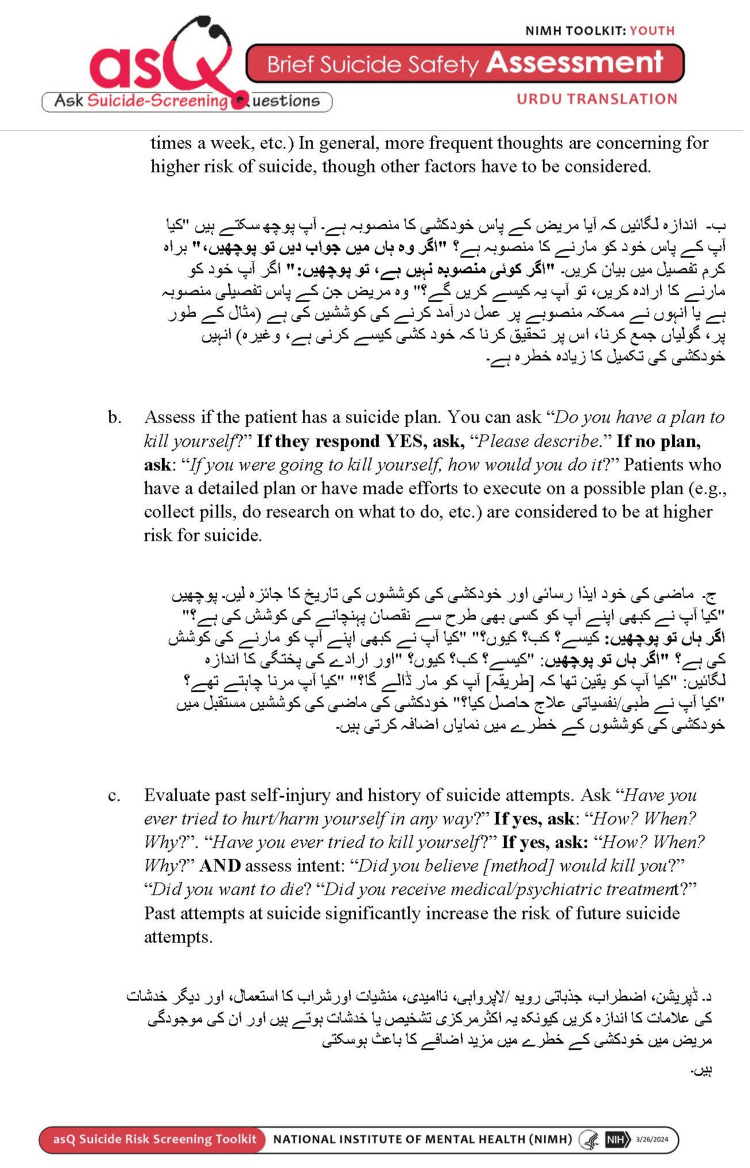
Brief Suicide Safety Assessment (BSSA) Urdu certified translation page 2.

**Figure 3:**
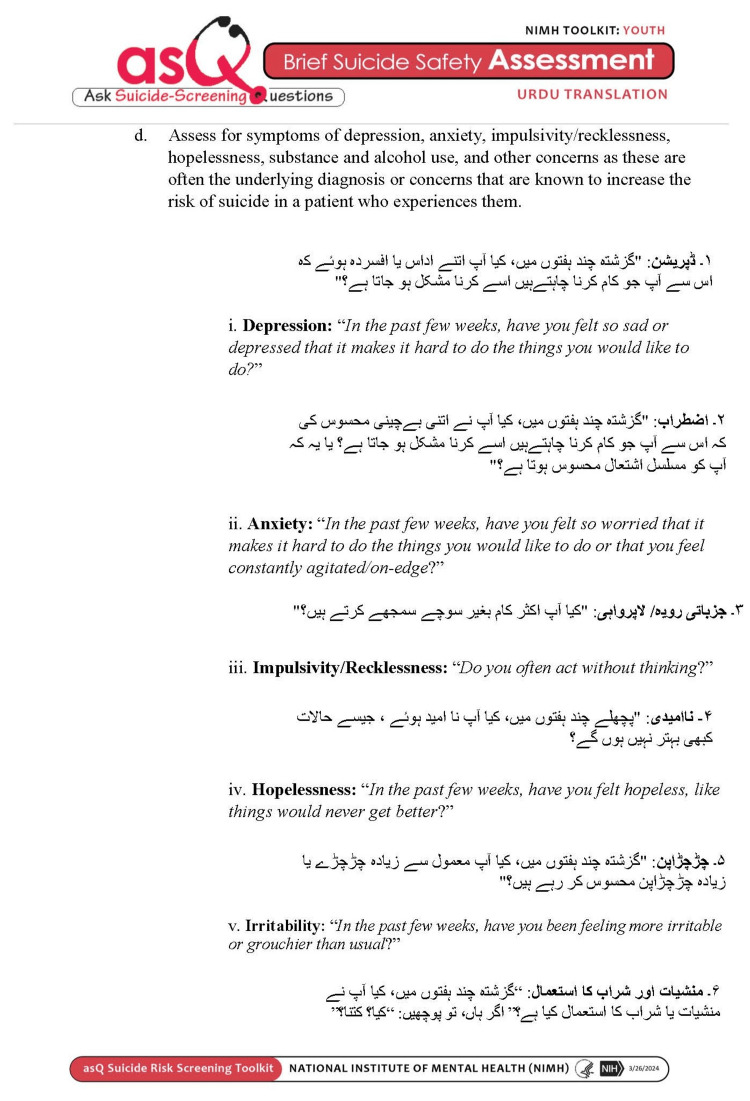
Brief Suicide Safety Assessment (BSSA) Urdu certified translation page 3.

**Figure 4:**
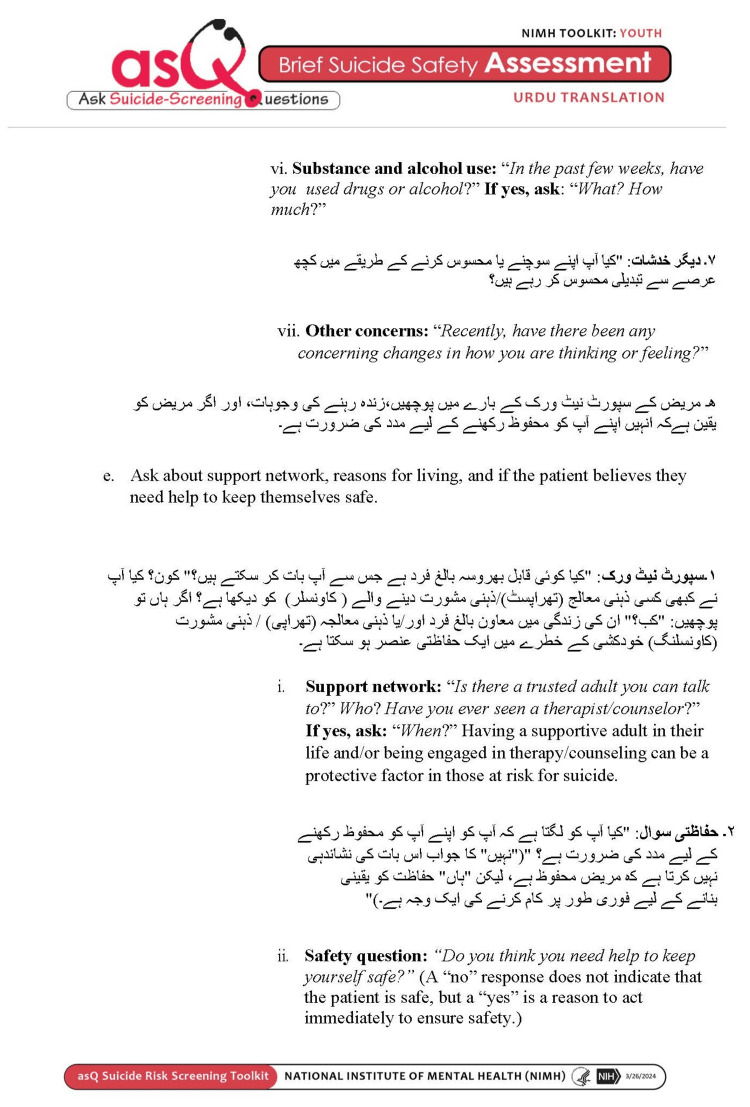
Brief Suicide Safety Assessment (BSSA) Urdu certified translation page 4.

**Figure 5:**
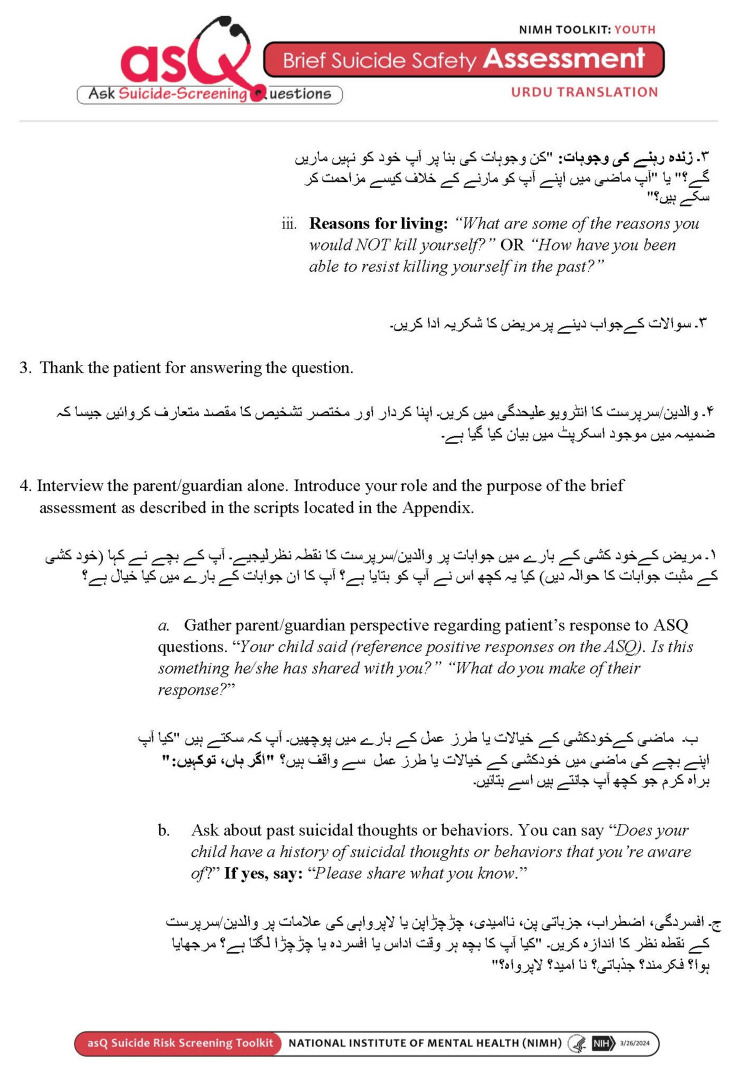
Brief Suicide Safety Assessment (BSSA) Urdu certified translation page 5.

**Figure 6:**
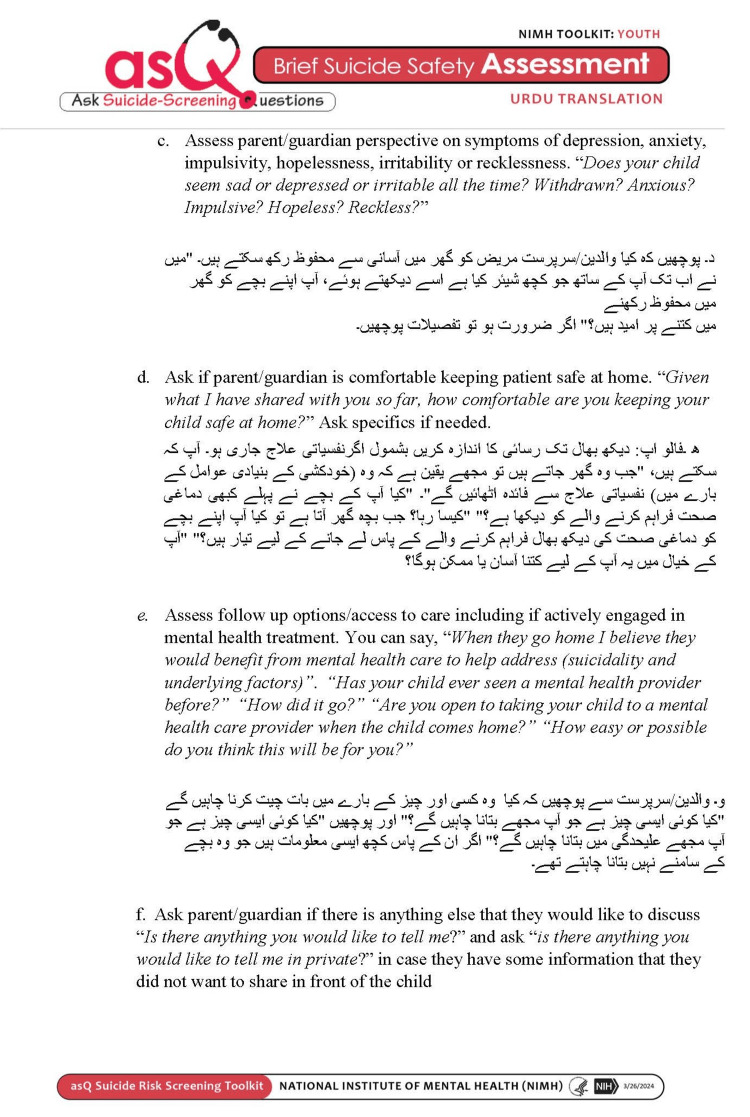
Brief Suicide Safety Assessment (BSSA) Urdu certified translation page 6.

**Figure 7:**
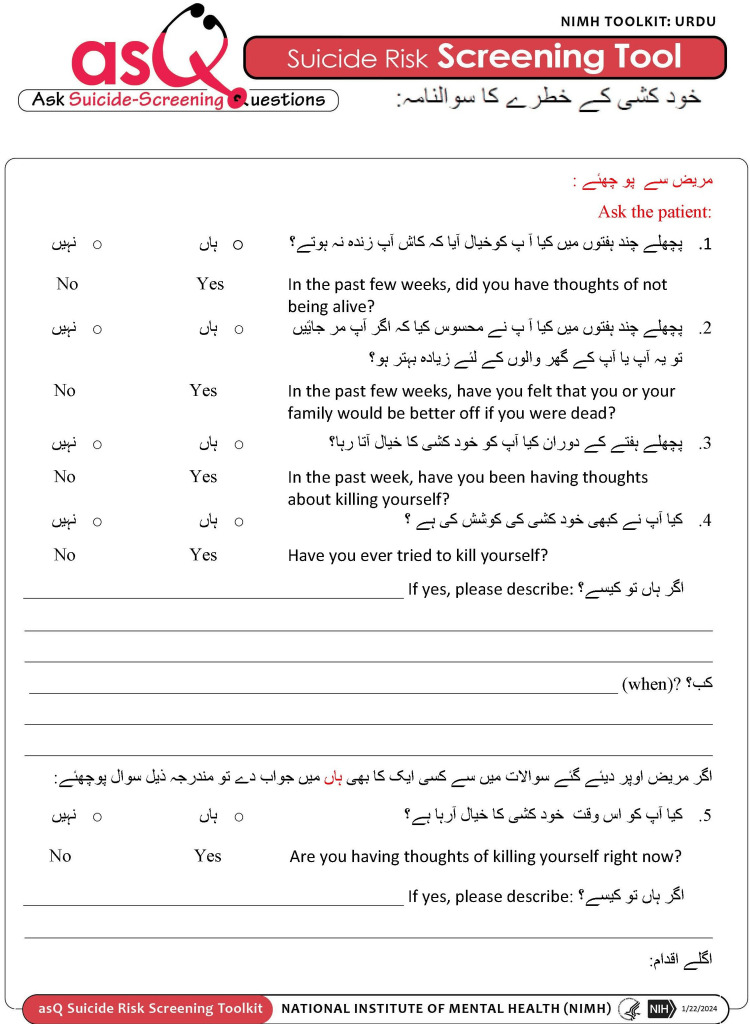
Ask Suicide-Screening Questions (ASQ) Urdu certified translation page 1.

**Figure 8:**
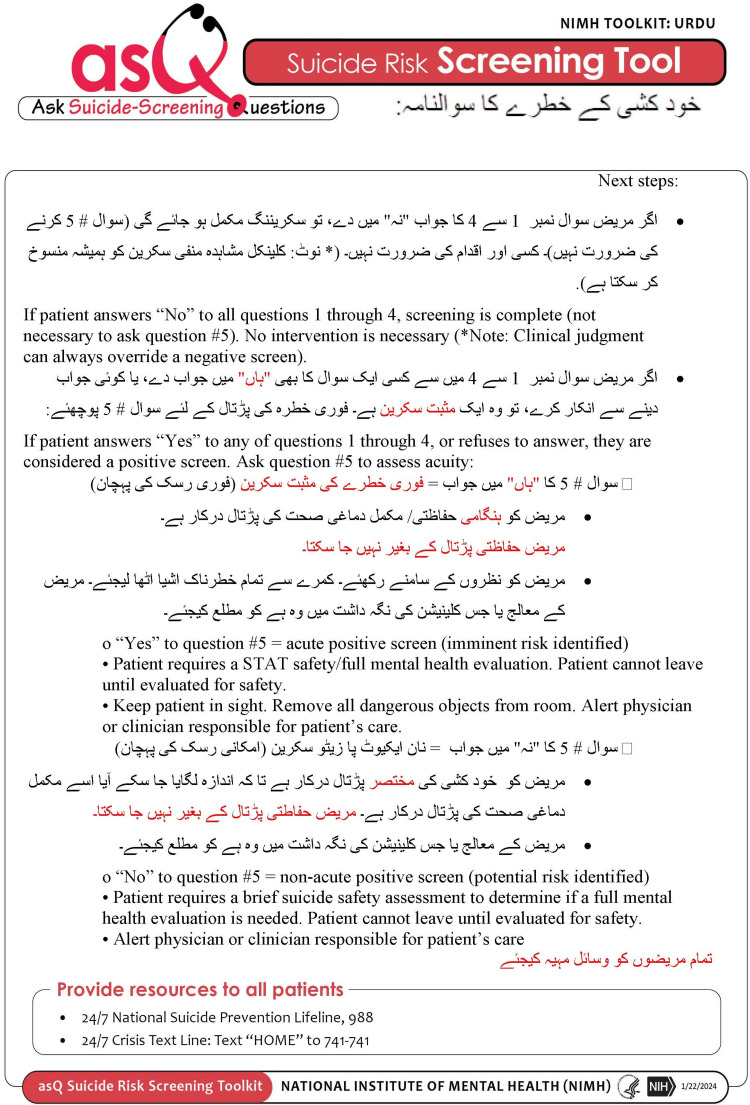
Ask Suicide-Screening Questions (ASQ) Urdu certified translation page 2.
